# Prenatal diagnosis of fetal congenital mesoblastic nephroma by ultrasonography combined with MR imaging: A case report and literature review

**DOI:** 10.1097/MD.0000000000024034

**Published:** 2021-01-22

**Authors:** Meiling Che, Fan Yang, Huilin Huang, Hongyang Zhang, Cong Han, Nao Sun

**Affiliations:** Department of Center for Reproductive Medicine and Center for Prenatal Diagnosis, The First Hospital of Jilin University, Changchun, Jilin, P.R. China.

**Keywords:** congenital mesoblastic nephroma, magnetic resonance imaging, prenatal diagnosis, ultrasonography

## Abstract

**Rationale:**

Fetal congenital mesoblastic nephroma (CMN) is a rare renal tumor, characterized by polyhydramnios, premature birth, and neonatal hypertension. In the prenatal stage, it is particularly difficult to diagnose CMN either by ultrasonography or magnetic resonance imaging (MRI). Thus, CMN is frequently detected in the third trimester in the clinical scenario.

**Patient concerns:**

A 29-year-old G2P0 pregnant woman took routine prenatal examinations in our hospital. The fetal right kidney abnormality was not observed after 2 systematical ultrasonic examinations (at 24 and 31 weeks of gestation respectively), and only an increase was noticed in the amniotic fluid index (from 19.3 to 20.8 cm).

**Diagnosis:**

CMN was detected by antenatal ultrasonography and MRI as a fetal right renal mass at 35 weeks of gestation in our hospital.

**Interventions:**

The pregnant woman was admitted at a gestational age of 38 weeks and 5 days due to alterations in renal function. Further, the pregnant woman was administered with “oxytocin” to promote delivery, and the neonate underwent a right nephrectomy on the 9th day after birth.

**Outcomes:**

The pathological examination confirmed a cellular type of right CMN. The neonate recovered well after operation without adjuvant treatment. During 6 months of follow-up, the neonate grew well and showed no signs of recurrence or metastasis.

**Conclusion:**

Polyhydramnios detected during prenatal examination required attention due to the risk of malformation of fetal urinary system. Prenatal ultrasonography combined with MRI could not only clearly identify the origin of the tumor, but also distinguish the correlation between the tumor and adjacent structures, thereby leading to early diagnosis and favorable prognosis.

## 1 Introduction

Neonatal renal tumors account for 7% of all neonatal tumors,^[1–3]^ and congenital mesoblastic nephroma (CMN) represents the most common neonatal renal benign tumor.^[4]^ CMN was initially described by Bolande *et al.* in 1967 as a benign renal tumor different from other types of renal tumors.^[5]^ It is also known as mesenchymal hamartoma, leiomyomatous hamartoma and fetal renal hamartoma.^[6]^ Due to the similarity between the images of CMN and Wilm's tumor, prenatal diagnosis rate of CMN is extremely low.^[7,8]^ Interestingly, accumulating data have revealed that the combination of ultrasonography and magnetic resonance imaging (MRI) enables antenatal detection of CMN.^[9–14]^ Herein, we report a case study of CMN diagnosed by prenatal ultrasonography combined with MRI, in an attempt to offer novel references for early diagnosis of CMN.

## 2 Case report

This case report has been approved by the Medical Ethics Committee of First Hospital of Jilin University (2020-371) and written informed consent was obtained from the patient and caregiver prior to the study.

A 29-year-old pregnant woman (G2P0) with natural pregnancy this time was recruited in this study. She had no unhealthy life history and denied the previous exposure to radioactive materials, chemical raw materials or pesticides. The results of Down's screening and oral glucose tolerance test showed no abnormality. Her spouse was healthy and had no bad habits.

In 2019, the woman underwent 2 systematic ultrasonic examinations respectively at 24 and 31 weeks of gestation. No abnormality was found in the right kidney of the fetus, and only an increase was identified in the amniotic fluid index (AFI, from 19.3 cm to 20.8 cm).

**Figure 1:**
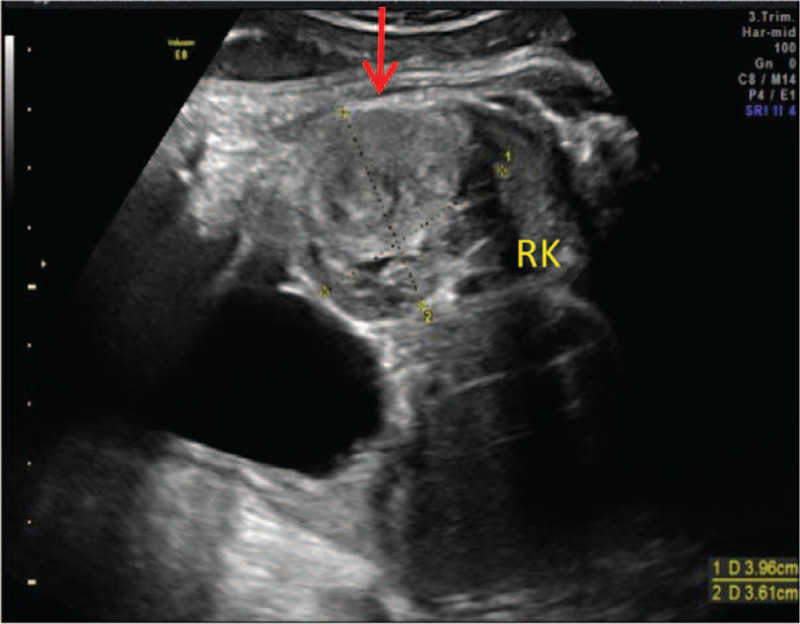
Prenatal ultrasonography detection indicates a 3 × 3 cm solid, well-defined, hyperechoic mass with uniform internal echo in the fetal right kidney.

**Figure 2:**
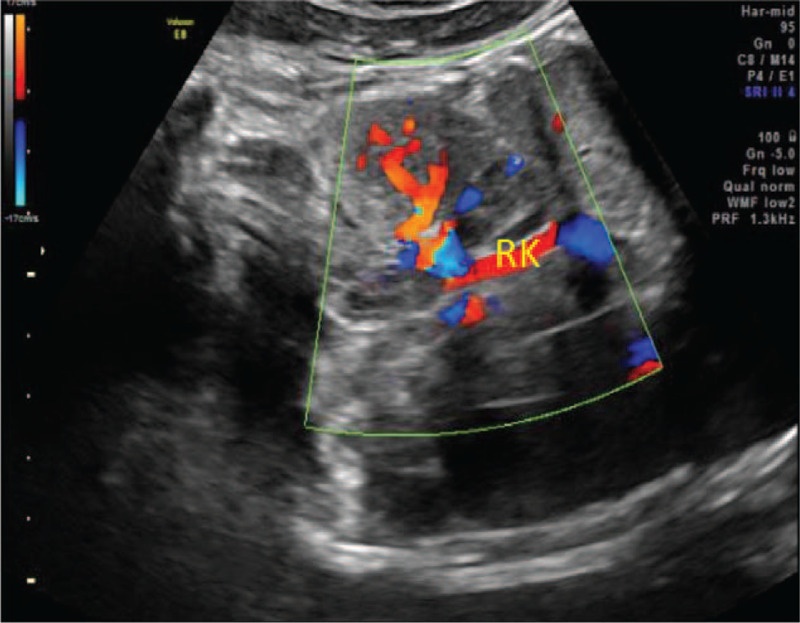
Color Doppler ultrasound shows a large nutrient vessel entering the inside of the tumor.

**Figure 3:**
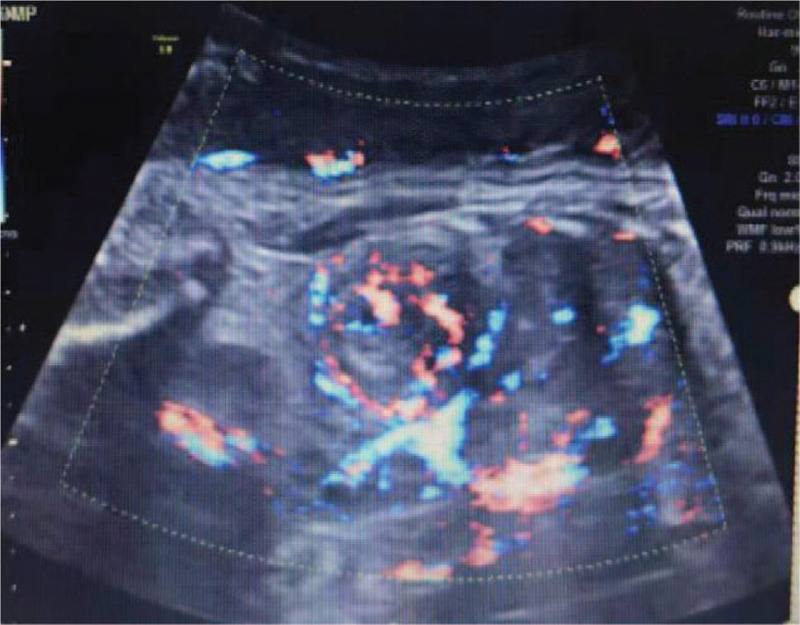
Representative images presenting circular blood flow signal around the tumor.

**Figure 4:**
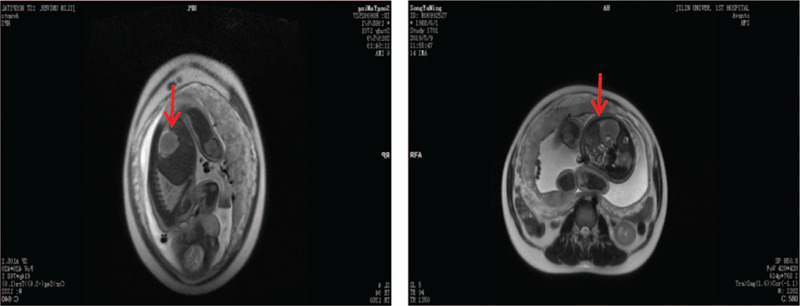
MRI shows that the posterolateral contour of the right kidney of the fetus is plump, locally convex, and the signals of TIWI, T2-haste and T2-trufi are similar to those of the renal parenchyma. The signals of DWI are slightly higher than those of the renal parenchyma, and the boundary between the lesion and the adjacent structures is clear. DWI = diffusion weighted imaging.

The woman was diagnosed with CMN through prenatal routine ultrasonography, which identified a fetal right renal mass at 35 weeks of gestation in the Center for Reproductive Medicine and Center for Prenatal Diagnosis in our hospital. The tumor was about 3.3 × 3.0 × 2.7 cm, which was solid, well-defined, and hyperechoic with uniform internal echo (Fig. 1). Moreover, color Doppler ultrasound presented a large nutrient vessel entering the inside of the tumor (Fig. 2), and circular blood flow signal could be seen around the tumor (Fig. 3) with an AFI of 25.3 cm. Based on observations through antenatal ultrasound, the condition was diagnosed as “fetal right renal tumor, with a possibility of CMN.” Then, MRI was performed for the fetus on the second day. The results of MRI (Fig. 4) showed that the posterolateral contour of the right kidney of the fetus was plump, locally convex, about 3.0 × 1.9 × 2.6 cm in size. The signals of TIWI, T2-haste and T2-trufi were similar to those of the renal parenchyma, while the signals of diffusion weighted imaging were slightly higher than those of the renal parenchyma, and the boundary between the lesion and the adjacent structures were clear. The prenatal MRI suggested “fetal right renal tumor, and CMN should be considered first”.

The mother was admitted at a gestational age of 38 weeks and 5 days due to alterations in renal functions and the obstetrician used “oxytocin” to promote delivery. A female baby weighing 3.25 kg was born vaginally and had an Apgar score of 5 at 1 minute. Subsequently, the conditions of the neonate were improved after 10 minutes of tracheal intubation resuscitation. Postnatal ultrasonography showed a solid, well-defined, heterogeneous mass of 3.9 × 3.0 × 3.8 cm in the posterior upper part of the right kidney of the neonate. Right nephrectomy was performed on the 9th day of birth (surgical excision of the right kidney, perirenal fat, a section of the ureter over). Intraoperative findings indicated that the tumor was located on the outside of the right kidney and fused with the kidney. The blood supply of the tumor was abundant, the capsule of the tumor was intact, and the tumor did not protrude out of the kidney. No tumor thrombus was observed in the renal vein and inferior vena cava, and no obvious enlarged lymph nodes were found around the kidney.

**Figure 5:**
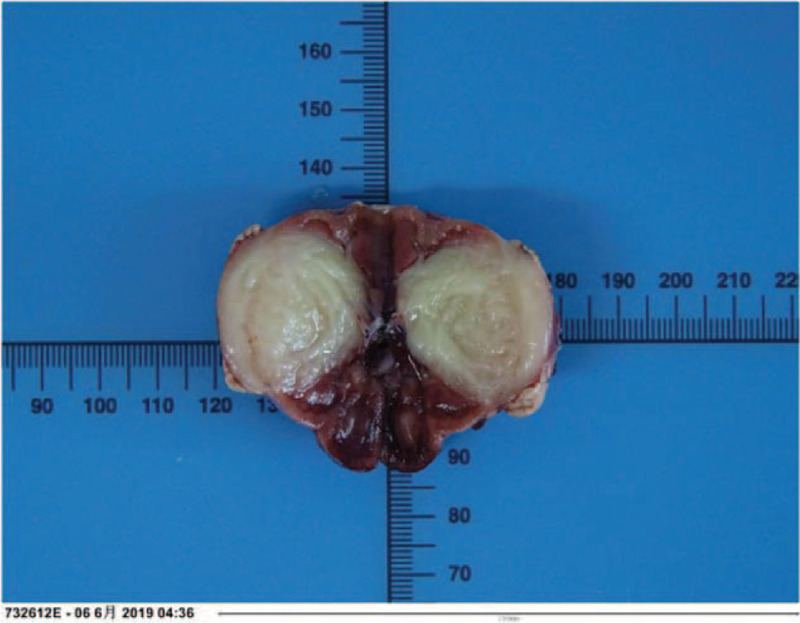
The tumor is adjacent to the renal hilum in the kidney, the cross section of tumor is toughness, solid with grey-white color, and the mass occupies most of renal parenchyma. No clear renal pelvis or calyces are found.

**Figure 6:**
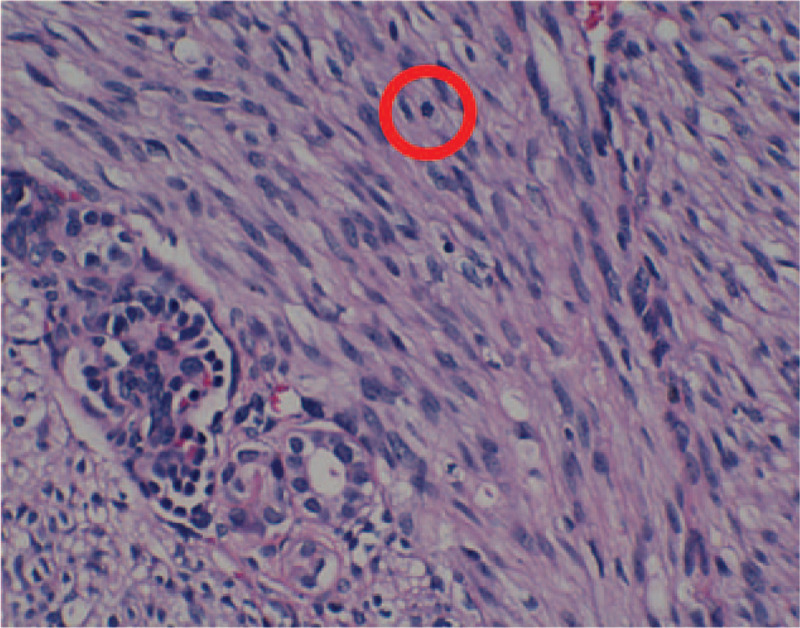
The tumor cells were spindle shaped and fibrous, with light staining of nucleus, rich cytoplasm and acidophilic, occasionally with mitotic image, and the tumor cells grow around tubules and glomerulus.

**Figure 7:**
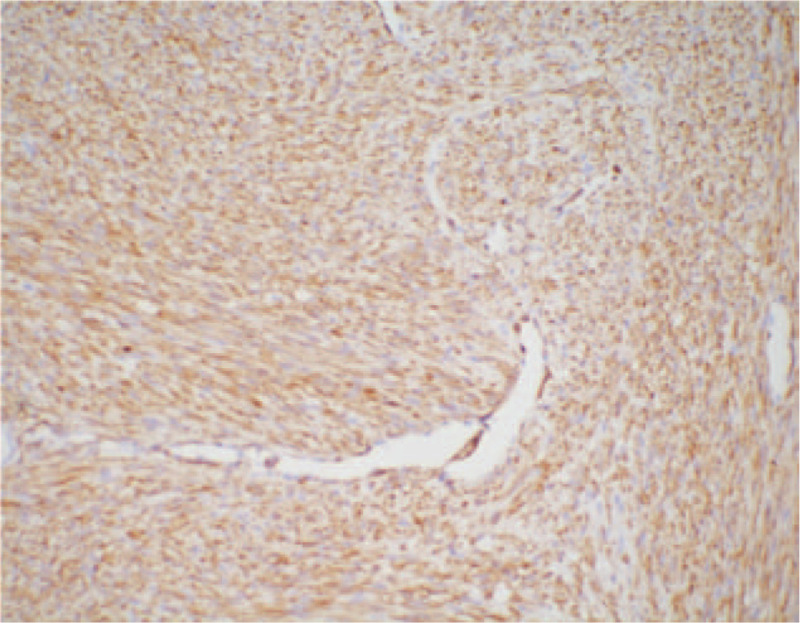
Actin shows weak positive expression in tumoral cells (magnification, x 200).

**Figure 8:**
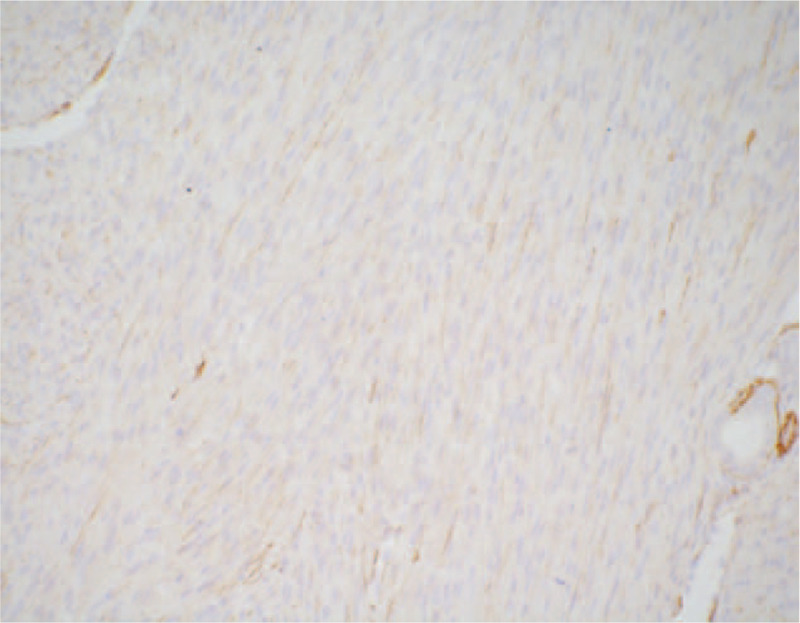
SMA shows positive expression in tumoral cells (magnification, x 200).

**Figure 9:**
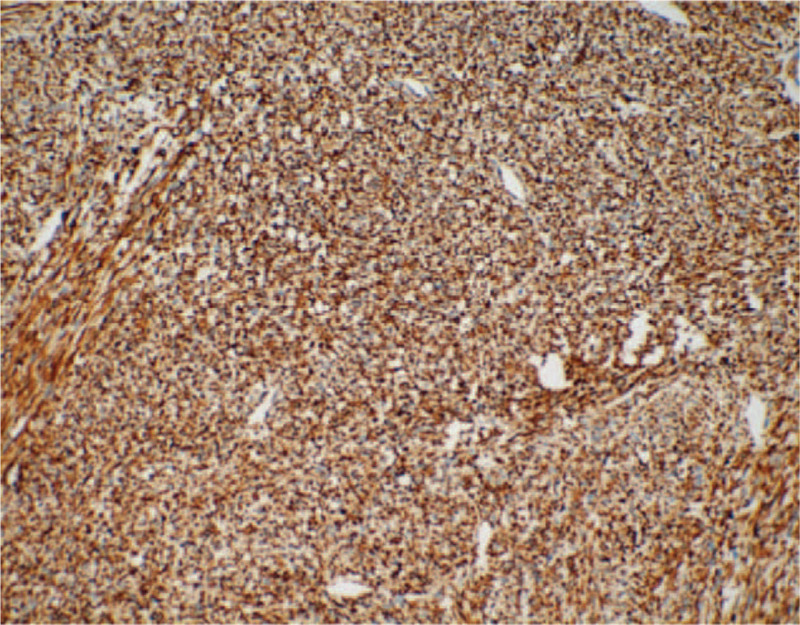
Vimentin shows positive expression in tumoral cells (magnification, x 200).

**Figure 10:**
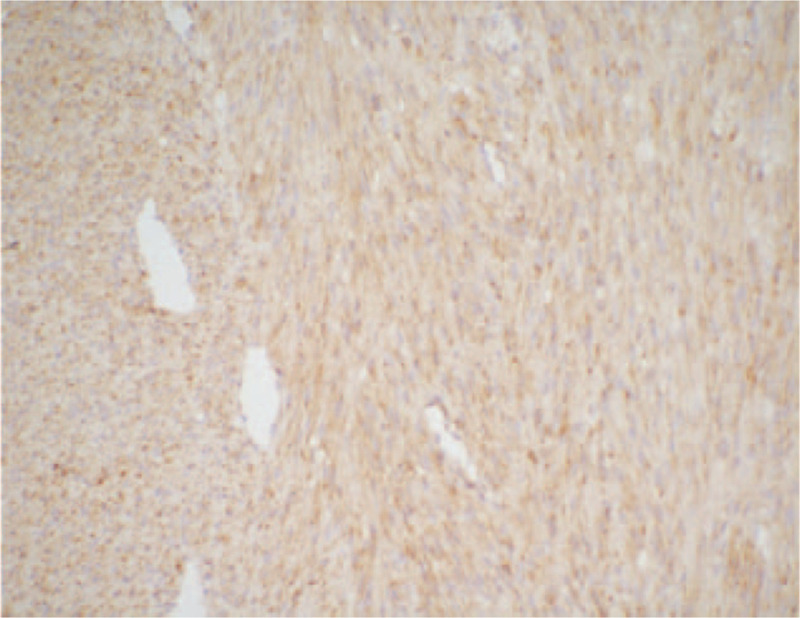
CD99 shows positive expression in tumoral cells (magnification, x 200).

**Figure 11:**
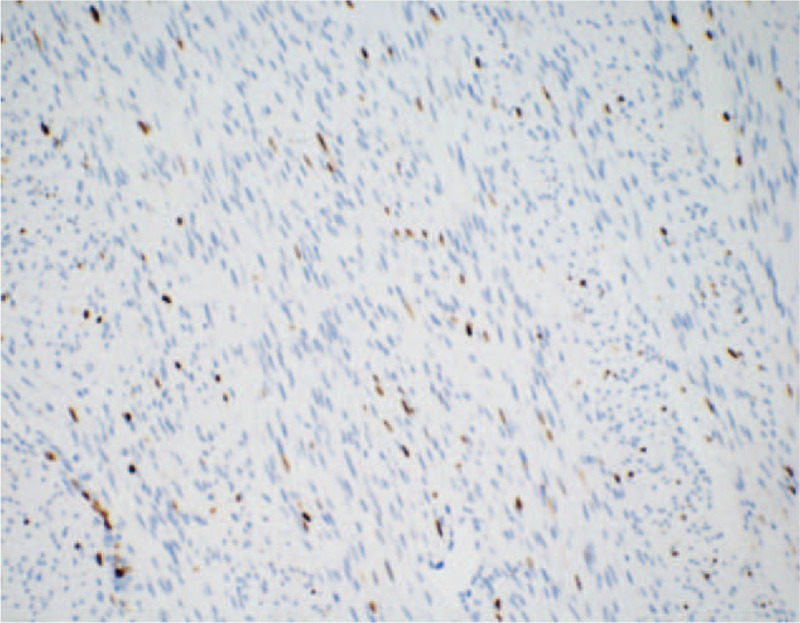
Ki-67 shows approximately 5% positive expression in tumoral cells (magnification, x 200).

According to macroscopic observations (Fig. 5), the volume of the right kidney was 4.5 × 3 × 3 cm, and there was a nodular mass of 3 × 3 × 2.8 cm adjacent to the renal hilum in the kidney. The cross section of tumor was tough and solid with grey-white color, the mass occupied most of renal parenchyma, and no clear renal pelvis or calyces were found. Another microscopic view (Fig. 6) indicated that the tumor cells were spindle shaped and fibrous, with light staining of nucleus, rich cytoplasm and acidophilic, occasionally with mitotic image; and there were tumor cells around tubules and glomerulus. Further, immunohistochemical analyses presented positive expression of Actin (Fig. 7), SMA (Fig. 8), Vimentin (Fig. 9), CD99 (Fig. 10), and Ki-67 (Fig. 11) in tumoral cells. Moreover, CD34, CKpan, Desmin, MyoD1, Bcl2, CgA, S-100, Syn, and WT-1 showed negative expression in tumoral cells. Results of postoperative histopathological examinations revealed the cellular type of right renal CMN.

The mother received no postoperative chemotherapy nor radiotherapy. The neonate recovered well after operation and was discharged 5 days later. No signs of recurrence or metastasis were observed during the 6-month follow-up and the neonate was well developed without any adverse effects.

## 3 Discussion

CMN is a rare renal benign tumor associated with polyhydramnios, prematurity, and neonatal hypertension.^[15]^ Although rarely seen, CMN is the most common benign tumor of the kidney in the infants.^[16]^ Owing to the similarity between the imaging findings of the CMN and Wilm's tumor, it is difficult to prenatally diagnose CMN.^[7,8]^ Meanwhile, almost all fetal CMN cases were found in the third trimester,^[4]^ when a rapid increase occurred in tumor size.^[17]^ In terms of epidemiology, it has been suggested that CMN is more likely to occur in males and in the right kidney.^[18]^ The conditions of the case reported in this study are basically consistent with findings provided in these literatures.

In spite of the considerable advancement in ultrasonography technology, only 15% of fetal renal tumors are prenatally identified.^[3]^ In ultrasound detection, classic CMN can be a homogeneous or heterogeneous, solid, hypoechoic renal mass with an echogenic rim (‘ring sign’), and color Doppler analysis may show a ring of venous and arterial waveforms with/without low vascularity. Commonly, CMN presents as a large, unilateral renal mass, where occasionally emerging in cystic areas.^[10,11]^ In our case, prenatal ultrasonography showed a 3.3 × 3.0 × 2.7 cm uniform internal echo, solid, well-defined, hyperechoic mass in the fetal right kidney. Moreover, color Doppler ultrasound result indicated a large nutrient vessel entering the inside of the tumor; and the signals of circular blood flow were detected around the tumor, consistent with previous literature report. Wilm's tumor was highly similar to CMN, commonly appearing as an unencapsulated tumor with a clearly defined capsule, characteristically invading the normal renal parenchyma. Areas of hemorrhage and necrosis may be detected within the mass.^[1]^ Prenatal ultrasonographic examination of CMN and Wilms’ tumor are similar and absolute distinction can solely be identified pathologically.^[19]^ Further, previous studies have indicated that a considerable number of masses initially considered as Wilms’ tumor were ultimately diagnosed as CMN in the postnatal period.^[17,18]^ Considering the particular difficulty in differentiating Wilm's tumor and CMN, a previous study suggested that fetal renal tumors found in the prenatal stage should be considered as CMN.^[18]^ Moreover, congenital adrenal neuroblastoma distinguishes itself from CMN for being separated from the kidney with obvious margin and a mixed echo structure of solid and cystic components. Prenatal diagnosis mainly depends on the observation of the asynchronous movement between lesion and kidney during fetal breathing.^[1]^

Currently, the fetal MRI technology is preferred by obstetricians since MRI can provide a better tissue contrast and a variety of imaging planes regardless of the fetal orientation,^[20]^ which makes it particularly useful in the case of oligohydramnios.^[14]^ Further, MRI has some advantages as compared with ultrasound, especially in the identification of urogenital and encephalic malformations.^[21]^ In fetal MRI, CMN is well defined, homogeneously solid, and tends to be isointense to normal renal parenchyma on T2-weighted imaging. Moreover, normal renal parenchyma and circulation system may be distorted by compression of the masses.^[10,22,23]^ In our report, the signals of TIWI, T2-haste and T2-trufi were similar to those of the renal parenchyma, while the signals of diffusion weighted imaging were slightly higher than those of the renal parenchyma, which were in agreement with evidences reported in previous literatures. In the MRI, the boundary between the lesion and the adjacent structures was clear, which provided a reference for postnatal surgery. In addition, MRI can also be used for the evaluation of metastases or recurrence.^[17]^

Polyhydramnios serves as a clue for the prenatal diagnosis of fetal congenital obstruction of the gastrointestinal tract.^[24]^ In the process of antenatal examination, doctors often pay attention to the malformation of fetal digestive system rather than fetal urinary system when polyhydramnios was observed. Polyhydramnios could be detected in approximately 70% of CMN cases.^[2]^ The cause of polyhydramnios is not clear so far, whereas 2 speculations have been proposed:^[12,17,25,26]^ a reduction in the absorption of amniotic fluid by fetal gastrointestinal tract, or an increase in the production of fetal urine. The former is mainly due to the compression and physical stimulation of the tumor, which results in the slowing down of fetal gastrointestinal peristalsis and the hindrance of the fetal swallowing amniotic fluid. The latter is mainly a result of the presence of fetal renal tumor, which leads to polyuria due to increased renal perfusion, and the consequence of hypercalcemia could induce polyuria. In our case, polyhydramnios was also witnessed (AFI 25.3 cm). However, there was also a rare case where CMN was associated with oligohydramnios.^[27]^ In this case, the neonate was diagnosed as CMN based on the detection of anuria, hypotension, hyperkalemia, and disseminated intravascular coagulopathy after birth. As expected, babies with oligohydramnios present a poorer prognosis than those with polyhydramnios.

Nephrectomy is the major therapeutic method for CMN.^[28]^ If CMN was prenatally diagnosed and suitable treatment was adopted after birth, the 5-year survival and overall survival rates of infants could reach 94% and 96%, respectively.^[17]^ Apart from the correlation between CMN and prematurity as well as neonatal hypertension, recurrence and metastases have also been reported after nephrectomy.^[17]^ However, in our case, there was no premature labor, and the neonate recovered well after operation. Moreover, there was no sign of hypertension, recurrence, or metastasis during the 6 months of follow-up visit, and the neonate was well developed without any side effects. We will continue to follow up and observe closely.

## 4 Conclusions

During prenatal examination, we should not only pay attention to the malformation of fetal digestive system, but also the fetal urinary system, especially in the presence of polyhydramnios. CMN should be considered firstly upon the occurrence of fetal renal tumor. Further, prenatal ultrasonography combined with MRI could not only identify the origin of the tumor more clearly, but also better distinguish the tumor from adjacent structures, providing valuable information for postnatal surgery. In this sense, the combination of ultrasonography and MRI could, to a large extent, facilitate early diagnosis, appropriate treatment, and satisfactory prognosis.

## Author contributions

**Data curation:** Meiling Che, Cong Han.

**Investigation:** Huilin Huang.

**Supervision:** Hongyang Zhang.

**Writing – original draft:** Fan Yang.

**Writing – review & editing:** Nao Sun.
